# Communication Skills in Toddlers Exposed to Maternal SARS-CoV-2 during Pregnancy

**DOI:** 10.3390/life14101237

**Published:** 2024-09-27

**Authors:** Enrico Apa, Nicole Carrie Tegmeyer, Concetta D’Adamo, Eleonora Lovati, Chiara Cocchi, Paola Allegra, Francesco Ostello, Daniele Monzani, Elisabetta Genovese, Silvia Palma

**Affiliations:** 1 Audiology Unit, Dipartimento di Scienze Cliniche e di Comunità, Dipartimento di Eccellenza 2023–2027, Fondazione IRCCS Ca’ Granda, Policlinico of Milan, 20122 Milan, Italy; enrico.apa@policlinico.mi.it (E.A.); concetta.dadamo@policlinico.mi.it (C.D.); 2 Otorhinolaryngology and Audiology Unit, Department of Medical and Surgical Sciences for Children and Adults, Azienda Ospedaliero-Universitaria of Modena, 41126 Modena, Italy; 326249@studenti.unimore.it (N.C.T.); 176683@studenti.unimore.it (E.L.); allegra.paola@aou.mo.it (P.A.); ostello.francesco@aou.mo.it (F.O.); elisabetta.genovese@unimore.it (E.G.); 3 Functional Recovery and Rehabilitation Unit, ASL CN2-Ospedale Michele e Pietro Ferrero, Verduno, 12060 Cuneo, Italy; ccocchi@aslcn2.it; 4 ENT, Department of Surgical Sciences, Dentistry, Gynaecology and Paediatrics, University of Verona, Borgo Roma Hospital, 37134 Verona, Italy; daniele.monzani@univr.it; 5 Audiology, Primary Care Department, AUSL of Modena, 41100 Modena, Italy

**Keywords:** hearing loss, SARS-CoV-2 infection, audiological follow-up, language development, COVID-19 infection, MacArthur–Bates Communicative Development Inventory Questionnaire

## Abstract

Studies about the effects of SARS-CoV-2 on pregnant women and children born to positive women are controversial with regard to possible inner ear-related damage but most of them do not detect the involvement of this virus in auditory function. However, only a few studies on long-term effects on language development are currently available because of the recent onset of the pandemic. The aim of this study was to investigate the impact of SARS-CoV-2 infection on perceptual and expressive abilities and the emerging development of communication in young children. To this purpose, the MacArthur–Bates Communicative Development Inventory—Words and Gestures form (CDI-WG), was administered to parents. In total, 115 children whose mother was infected by SARS-CoV-2 during pregnancy were enrolled in the study and evaluated at the Audiology Service of the Modena University Hospital. All children underwent Otoacoustic Emissions (OAE) at birth: 114/115 had a “pass” result bilaterally, while 1 case had a unilateral “refer” result. Overall, 110/115 newborns (95.65%) underwent audiological evaluation between 10–18 months of age. In 5/110 patients (3.6%), the Pure Tone Average (PTA) result was equal to 35 dB; one case had a hearing threshold of around 50 dB due to a bilateral effusive otitis media. A notable finding was the percentage of children with tubal dysfunction in both evaluations, within 2 months of age and around 12 months of age. Most children revealed normal hearing. The CDI-WG was completed by 56/115 families. The rate of children below the fifth percentile was 8.9% for sentences understood, 12.5% for words understood, and 5.4% for words produced. Concerning CDI-Gestures, only 2 children (3.6%) were below the fifth percentile. A structured audiological follow-up in association with the evaluation of communication skills of children appears fundamental, particularly in the years of maximum neuroplasticity. Long-term studies are still necessary to evaluate the possible consequences of the pandemic.

## 1. Introduction

The first years of children’s lives are the most important for the development of language skills, with their families playing a fundamental role in providing adequate tools [1,2]. Indeed, the emergence of pragmatic communication occurs in interaction with caregivers and leads gradually to acquiring language [3]. A very short time after postnatal exposure, infants are able to discriminate between different prosodic patterns [4]. The first step of language development is characterized by the so-called “prelingual phase” (from birth to about 12 months of age). In the early lingual phase (from 1 to 2.6 years of age), children show signs of word comprehension and start producing isolated words and short sentences. Children with developmental language disorders are at risk for not achieving proper cognitive and emotional evolution [5]. For these reasons, they should be identified as soon as possible [6].

A delay in language development may be a symptom of many disorders, such as hearing loss, autism, mental retardation, etc. Hearing loss is one of the most common congenital anomalies, with a prevalence of 1 to 3 of every 1000 newborns [7]. Early diagnosis is essential in order to assess a rehabilitation program as it has been evidenced that the size and depth of vocabulary knowledge in hearing-impaired toddlers is lower than in their hearing peers [8].

Several infections during pregnancy might cause congenital hearing loss in the newborns, Cytomegalovirus, Toxoplasma, Rubeola, Varicella Zoster, etc. (the so-called TORCH complex) [9]. Infection timing is an important variable: the sooner the mother becomes infected, the higher the risk of developing of alterations [10]. In recent years, it has been suggested that SARS-CoV-2 could have similar behavior. The SARS-CoV-2 virus is new to humans and studies about its effects on children born to positive women are controversial with regard to possible inner ear-related damage. Most of them do not find an early involvement in auditory function and exclude an increased incidence of tubal dysfunction in affected subjects [11,12,13,14,15]. Moreover, the association between prenatal SARS-CoV-2 exposure and infant neurodevelopment is still unclear. Despite advances in understanding human immunity to SARS-CoV-2 infection, few studies have defined the relationship between viral exposure in pregnancy and its immunologic impact on the mother and newborn [16]. The infection could negatively impact fetal brain development through transplacental transmission of SARS-CoV-2 or it could produce placental dysfunction and preterm birth [17]. A recent study in mice revealed long-lasting neurological and cognitive changes as a result of prenatal SARS-CoV-2 infection [18].

However, data on long-term effects on both hearing function and language development in this population are not currently available because of the recent onset of the pandemic.

The aim of this study was to investigate if SARS-CoV-2 infection could impact on perceptual and expressive skills and the emerging development of communication in young children exposed to the virus during pregnancy, also on the base of the trimester of infection. The study has been organized in two phases. Firstly, all newborns enrolled, whose mother had been infected by SARS-CoV-2 during pregnancy, underwent the newborn hearing screening (NHS) tests planned for babies with risk factors for hearing loss [19]. In this first step of the audiological surveillance program, parents were given the MacArthur–Bates Communicative Development Inventory—Words and Gestures form (CDI-WG) questionnaire to complete and return on the occasion of the next evaluation at the hospital service. It was expected to gather information about the development of children’s communication skills and language competencies [20]. Subsequently, children underwent audiological follow-up to 1 year of age. The results of the questionnaires and the outcomes of the audiological evaluations have been then analyzed and discussed.

## 2. Materials and Methods

This is an observational study based on the daily activity of a third-level hospital audiological service. Since the pandemic outbreak, all women who accessed the hospital to give birth underwent a nasopharyngeal swab to exclude a SARS-CoV-2 infection.

Every year, around 3000 babies are delivered at the Neonatal Unit. Children born between November 2021 and February 2023, whose mother was infected by SARS-CoV-2 during pregnancy, were enrolled in the study and evaluated at the Audiology Unit of the Modena University Hospital.

The exclusion criteria were syndromic features (e.g., atresia or fistula auris, facial dysmorphia, etc.), TORCH infections, meningitis, encephalitis, family history of hearing loss with suspected genetic transmission, administration of aminoglycosides or other ototoxic drugs for more than five days, hyperbilirubinemia treated with exchange transfusion, admission to neonatal intensive care unit (NICU) for more than five days, birth weight <1500 g, gestational age <28 weeks, Apgar score at 1′ or 5′ minutes <4, and uncompleted screening protocol.

### 2.1. Audiological Evaluation Procedures

According to the two-stage regional newborn hearing screening (NHS) program, all newborns underwent Otoacoustic Emissions (OAE) at the Neonatal Unit before discharge [21]. As SARS-CoV-2 was considered a potential risk factor for hearing loss in the first years of the pandemic, newborns underwent the NHS tests planned for babies with risk factors for hearing loss (within two months of age). The analysis of the cases was also made according to the trimester of infection.

All the included cases underwent a complete audiological evaluation, including Auditory Brainstem Responses (ABR), bilateral acoustic immittance test and acoustic reflex (AR) measurements, and otoscopy [19].

As usual, OAE results were binary for each ear, with the “pass” result indicating the presence of a cochlear response or “refer” result in the case of a repeated unclear unilateral or bilateral response.

Concerning ABR, threshold V wave identification ≤30 dB nHL without pathological delay of latency was considered indicative of normal results. Acoustic immittance tests were performed in order to exclude potential over-estimations of the auditory threshold caused by middle or external ear dysfunctions.

All the families were invited to repeat audiological evaluation at the age of one year. In this second evaluation, children underwent otoscopy, a visual reinforcement audiometry (VRA) by a two-channel diagnostic audiometer (Piano Plus VRA, Audiology and Balance, Inventis Srl, Padova, Italy), and an immittance test with AR measurements. The latter were conducted using a Madsen Zodiac device (Natus^®^ Medical Incorporated, Taastrup, Denmark) at a probe tone frequency of 1000 Hz. VRA is based on the orientation reflex toward a sound source, requires the cooperation of the child, and can be used when infants are able to turn their heads. In a few cases, due to a lack of compliance, newborns underwent Evoked (TEOAE) or Distortion Product Otoacoustic Emissions (DPOAE) [22]. A Madsen AccuScreen device (Natus^®^ Medical Incorporated, Taastrup, Denmark) was used for this task.

The Pure Tone Average (PTA) was computed considering 500 Hz, 1000 Hz, 2000 Hz, and 4000 Hz and the severity of sensorineural hearing loss was defined according to the ASHA classification: slight (16 to 25 dB), mild (26 to 40 dB), moderate (41 to 55 dB), moderately severe (56 to 70 dB), severe (71 to 90 dB), and profound (91 dB+) [23].

### 2.2. MacArthur–Bates Communicative Development Inventory—Words and Gestures Form

The MacArthur–Bates Communicative Development Inventory (CDI) is utilized to assess the child’s language development and general communication. It was designed to be completed by parents to collect early vocabulary and non-verbal communication. The Italian version was validated by Caselli and Casadio in 1995 [24]. In our department, the CDI questionnaire is routinely administered to all families of children who undergo audiological follow-up in the first years of life.

Given the strong changes that occur between the first and third years of life, it has been necessary to develop two forms: *Words and Gestures* (CDI-WG), designed for typically developing children ages 8–24 months, as a measure of emerging receptive and expressive vocabulary and the use of communicative or symbolic gestures, and *Words and Sentences* (CDI-WS), designed for typically developing children ages 16–30 months as a measure of developing expressive vocabulary and a number of aspects of early grammar development.

The form used in this study, Words and Gestures (CDI-WG), has three major parts. The first, *Early Words* includes three questions on children’s responsiveness to language (first signs of attention) and, subsequently, in a second section, using a list of 28 items, parents identify sentences that the child understands. The second part, called *Lexicon*, contains two questions on how often the child imitates or produces words spontaneously and, in the following section, a vocabulary checklist consisting of 408 items divided into 19 semantic categories: sound effects, animal names, vehicles, names, toys, food items, articles of clothing, body parts, furniture, household objects, places to go, people, games and routines, verbs, words for time, adjectives, pronouns, question words, prepositions, and quantifiers. For each item, the respondent indicates whether the child “understands” and/or “understands and says”.

The third part, called *Actions and Gestures* (AG), consists of 63 communicative and symbolic actions and gestures. Its subsections include First Communicative Gestures, Games and Routines, Actions with Objects, Symbolic Playing Pretending to be a Parent, and Imitating Other Adult Actions.

At the end of the questionnaire, there is a basic information sheet for collecting information on personal data, the child’s medical history, exposure to languages other than Italian, and parental education and occupation.

The speech therapist and the phoniatrician of the audiological service, co-authors of this paper, collected, as in usual practice, the questionnaires and compared the results to the validated reference values [24].

In order to maintain the reliability of the CDI-WG, the different sections of the questionnaire were explained to parents to ensure their understanding of the various assignments. Parents were advised to observe the child during playing and spontaneous interactions at home in the week before the compilation.

To calculate the total score for receptive and expressive vocabulary, the speech pathologists computed the number of words. which were correctly understood and pronounced.

The number of deictic and communicative gestures was also considered. Deictic gestures are essentially *pointing*, *showing*, and *giving*, which acquire meaning depending on the context. On the contrary, communicative gestures (*conventional* and *representational*) can be understood by anyone observing the toddler.

The number of Sentences Understood, Words Understood, Words Produced, and Gestures were expressed as percentiles and compared to the results obtained in the normative sample [24]. A child was considered communicatively fragile and sent for subsequent referral for results below the 5th percentile.

Considering the correct age for gestation, the Language Quotient in comprehension (LQ-C) and in production (LQ-P) were obtained by comparing the number of words understood or produced to the normative values representing the 50th percentile performance. Similarly, the Language Quotient in actions and gestures (LQ-G) was computed.

### 2.3. Data Analysis

All data were collected in a Microsoft Excel^®^ database (18.0) and anonymized. They were clinical data concerning gender, trimester of virus infection, outcomes of the audiological evaluations, and scores of the questionnaire (the number of words that were correctly understood and pronounced, the number of sentences understood, words understood, words produced, and the number of gestures) expressed as percentiles.

### 2.4. Statistics

The Statistical Package for Social Sciences (IBM SPSS^®^ (Armonk, NY, USA) version 25.0 for Microsoft Windows^®^, Redmond, Washington, USA) was used for statistical analysis and graphical representation. Clinical data regarding the participants were reported by descriptive statistics. Quantitative and qualitative variables were expressed as means (with standard deviations (SD) and rates, respectively).

The sample size was categorized into two independent sub-groups according to the trimester of SARS-CoV-2 maternal infection. In particular, an inferential analysis was performed considering newborns whose mothers were infected during the first trimester or those in whom the maternal infection occurred after the 12th week of pregnancy. Given the small size of the sample group and regardless of the distribution, Fisher’s exact tests were used to verify the association between categorical variables, while Mann–Whitney U-tests were used for continuous variables. The level of statistical significance was considered reached if the *p*-value (two-sided) was <0.05 in all procedures. All quantitative parameters of the CDI-WB were considered. The fifth percentile was considered as the cut-off value for Phrases Understood, Words Understood, Words Produced, and Gestures [24].

### 2.5. Ethical Issues

This study was conducted in accordance with the Declaration of Helsinki and the protocol was approved by the Ethical Committee of Modena (Protocol AOU 0010385/22). When the questionnaire was delivered to parents, they signed an informed consent on the purpose of the research.

## 3. Results

### 3.1. Audiological Evaluation

A total of 115 newborns were enrolled in the study, 50 were males (43.48%) and 65 were females (56.52%). The majority of children (104/115) were born in 2022. In 23 cases (20%), a maternal infection occurred in the first trimester, whereas in 92 cases (80%) it occurred in the second or the third trimester. The audiological features are reported in Table 1.

All children underwent OAE at birth, 114/115 had a “pass” result while one case had a unilateral “refer” result. Subsequently, ABR was performed in 55 newborns (47.82%), whereas 5 (4.35%) and 31 (26.96%) underwent DPOAE and repeated TEOAE, respectively. In 24 cases (20.87%), the audiological tests were deferred due to Eustachian tube dysventilation bilaterally or because of otitis media with effusion (OME). The case with unilateral refer showed an ABR threshold of around 40 dB in that ear.

Considering all the ears, in 106 cases (96.36%), the V wave threshold was ≤30 dB, whereas in 4 cases (3.64%), the threshold was determined around 40 dB due to effusive otitis media. Among these, in only one case, the PTA was equal to 35 dB and consistent with conductive hearing loss. In general, the mean value of the V wave threshold was 29.63 dB nHL (20–50 dB; SD ± 4.14 dB (see Table 1). In none of the cases considered were ABR, TEOAE, or DPOEA found to be bilaterally altered.

Overall, 110/115 newborns (95.65%) underwent audiological evaluation at the age of one year. Two newborns (1.74%) were not reliably tested using VRA and therefore, TEOAE was adopted, resulting in a ‘pass’ result bilaterally. A catarrhal infection with tubal dysfunction was bilaterally detected in the other 3 cases (2.61%) which had been considered normal-hearing children on the basis of the first audiological assessment.

In 5/110 patients (3.6%), the PTA resulted in equal to 35 dB and one case had a hearing threshold of around 50 dB due to a bilateral effusive otitis media. All the other children had a normal hearing threshold.

### 3.2. Communication Skills

Of the 115 children enrolled in the study, 56 CDI-WG questionnaires could be analyzed (48.7%) as many parents forgot to complete or return them. A few cases have been excluded from the analysis as poorly compiled. The mean age of the sample was 12.95 months (8–19 months; SD ± 2.51). Table 2 shows their distribution according to age and gender. Six children were exposed to bilingualism. The results of the evaluation of each section of the questionnaire are reported in Table 3.

Considering the whole CDI-Words section, the results of 15 (26.7%) children were inferior to the 5th percentile in at least one part of the subsection. This group was composed of 7 females and 8 males. Overall, 8 of them were aged 13 months or less, and the others were aged between 14 and 18 months. The rate of children below the 5th percentile was 8.9% for sentences understood, 12.5% for words understood, and 5.4% for words produced. One of these children was exposed to bilingualism, one had unilateral moderate hearing loss, and the others were distributed between 11 and 18 months of age. Concerning gesture production, only 2 children (3.6%) were below the 5th percentile.

**Figure 1:**
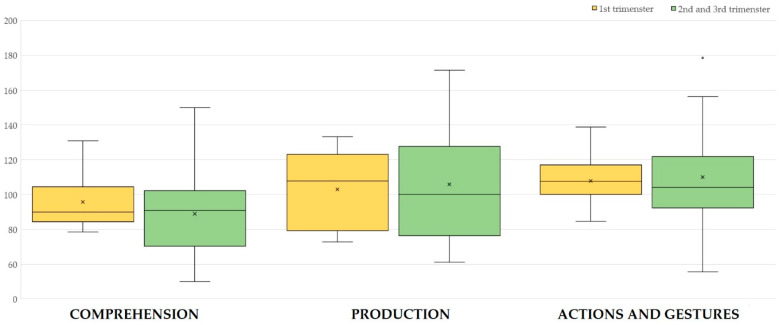
Language quotients of CDI-WG according to the trimester of maternal SARS-CoV-2 infection. Each box is included between the first and third quartile; the box’s height is equivalent to the inter-quartile range (IQR) and contains 50% of the measurements. Values that deviate from the box by more than 1.5 of IQR upward or downward are considered potential outliers and are represented with × or °.

The language quotients are represented in Figure 1. No significant differences were observed comparing newborns of mothers in which the infection occurred in the first trimester of pregnancy or later. In particular, using the Mann–Whitney U test, *p*-values resulted in 0.426 for LQ-C, 0.880 for LQ-P, and 0.932 for LQ-G.

## 4. Discussion

COVID-19 infection during pregnancy was not found to be a risk factor for hearing loss, according to the newborn hearing screening results [14,25,26] and the sample object of our study that confirmed these findings. Only one child that had a unilateral threshold around 40 dB continues to be under examination, as there could be other risk factors involved.

The trimester of maternal SARS-CoV-2 infection was irrelevant in results suggesting that the viral behavior is not similar to the TORCH complex one. A notable finding was the percentage of children with tubal dysfunction in both evaluations, within 2 months of age and around 12 months of age. In this regard, it has been recently reported that infection with COVID-19 may be associated with an increased risk of recurrent acute otitis media in children [27]. It seems that no matter the cause and the entity of hearing impairment, monitoring these children is essential.

Determining a hearing threshold is the first step to ensuring that children benefit from the opportunities to develop communication skills. As suggested by the Joint Committee on Infant Hearing, an early hearing detection program is essential, through a multidisciplinary approach and with trained personnel for such young children [19].

It is known that the process of language acquisition is tied to mutual and continuous interaction between brain development and the environment. Brain plasticity is maximum in the first years of life and allows children to implement progressive modeling of the neuronal circuits involved in the development of language. In consideration of the widespread impact of SARS-CoV-2 infections among pregnant women, understanding if there is an association between prenatal SARS-CoV-2 exposure and infant neurodevelopment is essential to assess the long-term consequences of the pandemic. This association is still unclear. In a study through the Bayley-III Scales of Infant and Toddler Development, it has been reported that children exposed to antenatal COVID-19 have a higher frequency of developmental delay compared to their peers [28]. On the other hand, a cohort study provided evidence that prenatal exposure to SARS-CoV-2 infection is not associated with differences in neurodevelopment between the ages of 5 and 11 months [29]. Ayed et al. concluded that there was no difference in neurodevelopmental outcomes at 18 months in children infected with SARS-CoV-2 compared with the controls [30]. A prospective study of 20 neonates who presented severe acute respiratory SARS-CoV-2 infection during the first wave of the pandemic showed no evidence of hearing impairment but showed, rather, an increased risk of developmental delays in expressive and receptive language skills at 18–24 months of age. The entity of these delays was found to be mild in most cases [31].

These controversial results confirm that there is still the need for data assessing the long-lasting impacts that the pandemic had on children’s language and longer follow-up studies are required.

The MacArthur–Bates questionnaire allows us to collect information about the evolution of communicative and linguistic skills of very young children. Different versions of the instruments have been adapted to over 100 different languages, making the CDI one of the most widespread instruments assessing child language [32].

The choice to adopt CDI in this study is primarily due to the routine utilization in audiological follow-up in our institution, but several families did not complete the questionnaire, around 49% of the global sample, showing a lack of compliance. We concluded that more effort should be made to improve awareness of follow-up visits among families.

In our study, 26% of children whose questionnaire was available were below the 5th percentile in at least one part of the Word section, with a range from 5 to 12%. This result could be due to different factors, such as the difficulties of parents in comprehending the words understood/produced or exposure to bilingualism. It has been reported that for the group of children with scores below the median on words produced, the reliability of the CDI may be lower [33]. Another concern is to assess language production at an age where, in addition to great variability even in the normative sample, verbal production is still emergent.

In other studies, percentages around 12% of toddlers who scored ≤10th percentile on CDI-Words and Sentences between 24 and 30 months of age have been reported. These children have been identified as late talkers [34,35]. Globally, the results of our study seem to indicate that parents’ observations were sufficiently reliable even for the purpose of research, though the subjective component of family members can never be excluded when answering such questionnaires.

Another reason could be tied to pandemic restrictions and the use of medical masks. These behaviors decreased the interaction of children with adults, reducing their exposure to external acoustic and visual stimuli [36]. Our sample is composed mainly of children born in 2022, a year that, in our country, still has pandemic limitations.

The outbreak of SARS-CoV-2 (COVID-19) has constrained families’ daily routines and determined an increase in the time spent watching TV or doing similar activities [37]. Moreover, nurseries in that period were not attended, thus reducing the interactions of children with peers.

This is confirmed by another recent study that compared a group of children born in 2019–2020 with an equal group of children born before 2012 using the Catalan adaptation of the questionnaire. Significant differences in vocabulary between pre- and post-Covid children were not evidenced, although there was a tendency for children with lower vocabulary levels to be in the post-Covid group [38]. A large study along various continents has recently reiterated that children who hear more talk from adults demonstrably produce more speech [39]. Indeed, a recent review indicates that the neonatal brain at birth is ready to acquire language quickly from everyday input [40].

Traditionally, the questionnaires are completed on paper but today, there are other possibilities such as electronic or online format on a laptop/or smartphone. Electronic administration has some advantages as it does not require face-to-face contact and reduces the chance of lost forms. Mainly, scoring can be simplified since responses need not be transferred from the paper into an electronic format [41].

Good rates were obtained in gesture communication. The evaluation of the number of deictic gestures added important information about perceptive and communicative skills as they are positively correlated with the emergence of first words and are a good predictor of the development of verbal comprehension at 8–18 months and verbal production at 10–23 months [24,42].

Recently, a review of the evidence assessing the use of the CDI-WG as a screening tool pointed out an overestimation of the reliability of this instrument [43] but in our daily practice, the questionnaire is a part of the medical evaluation together with audiological tests. Results are always considered with circumspection and are interpreted by both a speech therapist and a phoniatrician.

The study has several limitations. The main limitation is that the number of newborns enrolled is limited. Actually, it was difficult to establish an accurate number of newborns from mothers infected by SARS-CoV-2 during pregnancy “a priori”. Many infections were asymptomatic or pauci-symptomatic, in a population that was largely immunized due to previous infections or vaccinations. Secondarily, only half of the families enrolled returned the compiled questionnaire. Moreover, there is an absence of a control group that should be represented by children with no other risk factors for hearing loss. In this regard, we compared the results from the questionnaire to the normative values. Anyhow, it has been already reported that it is difficult to indicate a gold standard in language delay [44].

Finally, in a study like this, based on clinical routine activity, the use of other scales, usually reserved for preterm children with a motor delay, could not be programmed.

## 5. Conclusions

As a novel disease with many unknowns relating to management and prognosis, COVID-19 remains a challenge, also for professionals involved in evaluating communicative skills in toddlers. The routine use of the MacArthur–Bates questionnaire, in the presence of a complete hearing function evaluation, is useful to collect information about the evolution of communicative and linguistic skills of very young children but more effort should be made to improve awareness of follow-up visits among families. A notable finding was the percentage of children with tubal dysfunction in both evaluations, within 2 months of age and around 12 months of age.

Even though we do not have evidence that this virus is a specific risk factor for language disorder, long-term studies about children’s neurodevelopment are necessary to determine the possible consequences of the pandemic. A structured audiological follow-up together with the evaluation of the communication skills of children appears necessary, particularly in the years in which neuroplasticity is maximum.

## Figures and Tables

**Table 1:** Audiological features according to the trimester of maternal SARS-CoV-2 infection.

	Total	1st Trimester	2nd and 3rd Trimester	*p*-Value
Sample size	115	23 (20%)	92 (80%)	-
Gender	50 males (43.48%) 65 females (56.52%)	9 males (43.48%) 14 females (56.52%)	41 males (44.57%) 51 females (55.43%)	0.815 ^a^
**NHS—Evaluation at 2 Months** **of Age**				
OAE at birth	229 (99.57%) pass	46 (100%) pass	183 (99.46%) pass	0.995 ^a^
ABR	n = 55 29.63 dB nHL (20–50; ±4.14)	n = 5 28.50 dB nHL (20–30; ±3.38)	n = 50 29.74 dB nHL (20–50; ±4.21)	0.328 ^b^
TEOAE	n = 31 26 (83.87%) p/p	n = 9 8 (88.89%) p/p	n = 22 18 (81.81%) p/p	0.732 ^a^
DPOAE	n = 5 5 (100%) p/p	n = 3 3 (100%) p/p	n = 2 2 (100%) p/p	1.000 ^a^
catarrhal patterns	n = 24	n = 6	n = 18	1.000 ^a^
**Audiological Evaluation at 12–18 Months of Age**				
Age	12.62 months (7–19; SD ± 2.46)	11.97 months (7–18; SD ± 2.14)	12.78 months (7–19; SD ± 2.52)	0.102 ^b^
VRA	n = 110 26.09 dB nHL (20–50; SD ± 4.51)	n = 22 27.05 dB nHL (20–50; SD ± 6.11)	n = 88 25.85 dB nHL (20–35; SD ± 4.03)	0.269 ^b^
TEOAE	n = 2 2 (100%) p/p	n = 0 -	n = 2 2 (100%) p/p	1.000 ^a^
catarrhal patterns	n = 3	n = 1	n = 2	1.000 ^a^

^a^ Fisher’s exact test, ^b^ Mann–Whitney U test. Abbreviations: p/p = pass/pass; SD = standard deviation.

**Table 2:** Distribution of cases by age and gender.

Age (In Months)	Males	Females	Total
8	0	1	1
10	1	2	3
11	5	12	17
12	5	5	10
13	5	1	6
14	4	3	7
15	1	0	1
16	2	2	4
17	0	3	3
18	1	1	2
19	0	2	2
Total	24	32	56

**Table 3:** CDI-WG findings according to the trimester of maternal SARS-CoV-2 infection.

	Total	1st Trimester	3rd Trimester	*p*-Value
Sample size	56	14 (25%)	42 (75%)	-
Gender	24 males (42.90%) 32 females (57.10%)	5 males (35.71%) 9 females (64.29%)	19 males (45.24%) 23 females (54.76%)	0.730 ^a^
Age	12.95 months (8–19; SD ± 2.51)	12.14 months (10–18; SD ± 2.07)	13.21 months (8–19; SD ± 2.61)	0.138 ^b^
**CDI-Words**				
Phrases Understood	5 < 5th percentile (8.9%)	0 < 5th percentile (0.0%)	5 < 5th percentile (11.9%)	0.201 ^a^
Words Understood	7 < 5th percentile (12.5%)	1 < 5th percentile (7.1%)	6 < 5th percentile (14.3%)	0.437 ^a^
Words produced	3 < 5th percentile (5.4%)	0 < 5th percentile	3 < 5th percentile (7.1%)	0.408 ^a^
**CDI-Gestures**				
Gestures	2 < 5th percentile (3.6%)	0 < 5th percentile	2 < 5th percentile (4.8%)	0.398 ^a^
Deictic gestures	3.70 (1–4; SD ± 0.71)	3.71 (2–4; SD ± 0.61)	3.69 (1–4; SD ± 0.75)	0.570 ^b^
Communicative gestures	4.27 (1–8; SD ± 2.02)	4.07 (2–8; SD ± 1.77)	4.33 (1–8; SD ± 2.11)	0.881 ^b^
Total gestures	30.66 (8–57; SD ± 12.32)	28.71 (9–57; SD ± 12.02)	31.31 (8–57; SD ± 12.49)	0.823 ^b^

^a^ Fisher’s exact test, ^b^ Mann–Whitney U test. Abbreviations: CDI-WG: MacArthur–Bates Communicative Development Inventory—Words and Gestures Form.

## Data Availability

Raw data were generated at the Azienda Ospedaliero-Universitaria of Modena. Derived data supporting the findings of this study are available from the corresponding author on request.
